# Low-frequency repetitive transcranial magnetic stimulation can alleviate spasticity and induce functional recovery in patients with severe chronic stroke: A prospective, non-controlled, pilot study

**DOI:** 10.1016/j.heliyon.2023.e15564

**Published:** 2023-04-18

**Authors:** Yoshihiro Yukawa, Sumiya Shibata, Satoko Koganemaru, Masatoshi Minakuchi, Ryota Shimomura, Kazuhito Nakamura, Tatsuya Mima

**Affiliations:** a Department of Rehabilitation, Wakayama Professional University of Rehabilitation, 3-1, Minatohon-machi, Wakayama-shi, Wakayama, ZIP: 640-8222, Japan; b Department of Physical Therapy, Niigata University of Health and Welfare, 1398 Shimami-cho, Kita-ku, Niigata-shi, Niigata, Japan (ZIP: 950-3198); c Institute for Human Movement and Medical Sciences, Niigata University of Health and Welfare, 1398 Shimami-cho, Kita-ku, Niigata-shi, Niigata, ZIP: 950-3198, Japan; d Department of Regenerative Systems Neuroscience, Human Brain Research Center, Graduate School of Medicine, Kyoto University, 54, Shogoin, Kawahara-cho, Sakyo-ku, Kyoto-shi, Kyoto, ZIP: 606-8507, Japan; e Clover Care Medical Co.Ltd.,Wakayama, Japan, 2-34-17, Takao, Tanabe-shi, Wakayama, ZIP:646-0028, Japan; f Department of Rehabilitation, Murata Hospital, Osaka, Japan, 4-2-1, Tajima, Ikuno-ku, Osaka-shi, Osaka, ZIP: 544-0011, Japan; g Department of Neurosurgery, Murata Hospital, Osaka, Japan, 4-2-1, Tajima, Ikuno-ku, Osaka-shi, Osaka, ZIP: 544-0011, Japan; h Interdisciplinary Laboratory for Advanced Medical Science, Louis Pasteur Center for Medical Research, Kyoto, Japan, 103-5, Tanakamonzen-cho, Sakyo-ku, Kyoto-shi, Kyoto, ZIP: 606-8225, Japan; i Graduate School of Core Ethics and Frontier Sciences, Ritsumeikan University, 56-1, Tojiin, Kitamachi, Kita-ku, Kyoto-shi, Kyoto, ZIP: 603-8577, Japan

**Keywords:** Interhemispheric inhibition, Occupational therapy, Stroke, Transcranial magnetic stimulation, Upper limb hemiparesis

## Abstract

**Objective:**

Developing new therapies to improve motor function in patients with severe chronic stroke remains a major focus of neurorehabilitation. In this prospective, non-controlled, pilot study, we aimed to investigate the effects of low-frequency repetitive transcranial magnetic stimulation (rTMS) combined with occupational therapy (OT) on the motor function recovery of the affected upper limb in chronic stroke patients with severe upper limb hemiparesis.

**Methods:**

Consecutive patients (n = 40) diagnosed with chronic stroke (time since stroke, ≥1 year) and upper limb hemiparesis were enrolled in this study. Patients were classified according to the Brunnstrom recovery stage (BRS) for fingers. The severity of upper limb hemiparesis was categorized as mild (BRS IV–VI) or severe (BRS I–III). Patients received low-frequency rTMS to the contralesional primary motor area (M1) followed by OT for 12 consecutive days. The primary outcome was upper limb motor recovery, as measured with the Fugl-Meyer assessment (FMA). Secondary outcomes included manual dexterity, upper limb use, spasticity of the fingers and wrist, and motor evoked potential (MEP).

**Results:**

Patients with severe hemiparesis showed a significant increase in upper limb use, significantly improved quality of movement, and significantly reduced spasticity. Those with mild hemiparesis showed significant improvements in the FMA scores and manual dexterity, a significant increase in upper limb use and MEP, and significantly reduced spasticity.

**Conclusions:**

Low-frequency rTMS applied to the contralesional M1 combined with OT was effective in the rehabilitation of chronic stroke patients with severe upper limb hemiparesis by reducing the spasticity of the fingers.

## 1 Introduction

Thrombolysis and thrombectomy are breakthroughs in acute stroke treatment. Although thrombolysis and thrombectomy improve functional outcome [1,2], only a small few stroke patients are given to these treatments in some regions [3], and many stroke survivors do not fully recover from neurological deficits [4]. Since chronic upper limb impairments lead to loss of independence in daily life, developing effective therapies for chronic stroke is strongly required [3].

Repetitive transcranial magnetic stimulation (rTMS), a non-invasive brain stimulation technique, has been investigated as a potential tool for stroke rehabilitation, and its application to chronic stroke rehabilitation has been reported to have beneficial results [5]. One promising strategy is to decrease pathologically enhanced transcallosal inhibition in patients with chronic stroke through the application of inhibitory low-frequency rTMS on the contralesional hemisphere. This is based on the idea that the interhemispheric inhibition (IHI) between homonymous primary motor areas (M1) [6] influences motor performance. In a healthy brain, IHI can suppress a mirrored movement of the contralateral limb that is unfavorable for task performance [7]. The balance between bilateral M1s is disturbed in stroke patients. Because a stroke lesion decreases the excitability in the ipsilesional M1, the IHI from the ipsilesional M1 to the contralesional M1 is reduced, leading to the hyperexcitability of the contralesional M1. This results in the abnormally enhanced IHI from the contralesional M1 [8], which then leads to impaired functional recovery of the ipsilesional M1. Previous studies demonstrated that suppressing the overactivity of the contralesional M1 using low-frequency rTMS alone [[9], [10], [11]] or in combination with occupational therapy (OT) [[12], [13], [14], [15]] improves the motor function of the affected upper limb in patients with mild to moderate chronic stroke. However, whether the strategy is useful for patients with severe chronic stroke remains unclear.

The purpose of this prospective, non-controlled, pilot study was to investigate the effects of low-frequency rTMS with OT on the functional recovery of the affected upper limb in chronic stroke patients with severe hemiparesis.

## 2 Methods

### 2.1 Patients

The sample size of this study was calculated using G*Power [16], assuming an effect size of 0.5, a statistical power of 0.5, and an α level of 5% (based on a two-sided *t*-test indicating the difference between two dependent means). The calculated sample size was 18 patients.

**Fig. 1:**
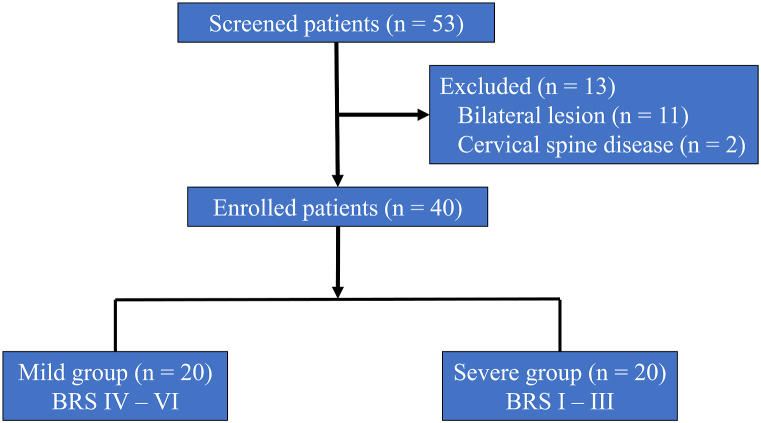
Flow chart of the pilot study.

Fifty-three consecutive patients with chronic stroke (time since stroke, ≥1 year) who presented to our hospital for neurorehabilitation over a period of 6 years were included according to the following criteria: men and women aged 19–90 years, presence of upper limb hemiparesis without spontaneous recovery, absence of higher cognitive impairment, absence of active physical or psychiatric illness, absence of recent (within 1 year) history of seizure, absence of epileptogenic discharges in pretreatment electroencephalogram among patients with a past history of seizure, and absence of contraindications for TMS [17]. Eleven patients with bilateral stroke lesions and two patients with cervical spine disease were excluded. A total of 40 patients were enrolled in this study (Fig. 1). The severity of upper limb hemiparesis was categorized according to the Brunnstrom recovery stage (BRS) for fingers as mild (BRS IV–VI; deviations from synergy) or severe (BRS I–III; no deviations from synergy) (Table 1). All patients took some medication [antispasticity medication, other neurological medications (antidepressant, antiepileptic, centrally acting analgesic, sedative, and sleep medication), peripherally acting analgesic, antiallergic, anticoagulant, antidiabetic, antifungal, antihypercholesterolemic, antihypertensive, antihyperuricemic, antiosteoporotic, antiplatelet, antitussive, antiuremic, branched-chain amino acid granule, bronchodilator, expectorant, gastrointestinal medication, iron, kudzu extract, laxative, steroid, thyroid hormone, urinary medication, vasodilator, and vitamin] except for two patients with mild and severe upper limb hemiparesis who did not have medication-related information. Two patients with mild upper limb hemiparesis took antispasticity medication, whereas none of the patients with severe upper limb hemiparesis took it. Ten and seven patients with mild and severe upper limb hemiparesis, respectively, took other neurological medications.

**Table 1:** Patient characteristics.

	Total	Mild group	Severe group	*p*
Women (%)	45	35	55	0.341
Age (y), mean ± standard deviation	61.9 ± 9.4	61.5 ± 10.5	62.4 ± 8.4	0.925
Time since stroke (d), median [interquartile range]	1243 [809–3077]	1366 [699–2291]	1208 [968–4710]	0.429
Type of stroke				0.010
Hemorrhage (%)	57.5	35	80	
Infarction (%)	42.5	65	20	
Lesion location (n)				0.731
Cortical/subcortical	12/28	5/15	7/13	
Side of stroke (n)				0.341
Left/right	22/18	9/11	13/7	
Medication (n)^a^				
Antispasticity medication		2	0	0.486
Other neurological medication		10	5	0.184
Brunnstrom recovery stage for hand-fingers (n)				
		Stage IV:12	Stage I:0	
		Stage V:8	Stage II:5	
		Stage VI:0	Stage III:15	

a Two patients with mild and severe upper limb hemiparesis, who did not have medication-related information, were excluded.

The protocol of the study was approved by the Clinical Research Ethics Committee of Murata Hospital (2011–001). Written informed consent was obtained from all patients.

### 2.2 Intervention protocol

All patients received 22 treatment sessions of low-frequency rTMS followed by 60-min OT for 12 consecutive days. They had two sessions a day from the 1st to 5th days and from the 8th to 12th days, and one session a day on the 6th and 7th days. They had another 60-min OT session without low-frequency rTMS on the 6th and 7th days.

A 70-mm figure-of-eight air-cooled coil and a Magstim Super Rapid^2^ rTMS stimulator (Magstim, Whitland, UK) were used to deliver rTMS (1 Hz) to the contralesional M1 of the first dorsal interosseous (FDI) muscle. The stimulation site was initially localized using stereotactic registration to each patient's structural magnetic resonance imaging scan with Brainsight (Rogue Research, Montréal, Canada). One session of low-frequency rTMS consisted of 1300 pulses at 90% resting motor threshold (rMT) of the FDI on the unaffected side. The rMT was calculated as the minimum stimulator output required to elicit a motor evoked potential (MEP) > 50 μV peak-to-peak amplitude in at least 5 of 10 consecutive trials [18].

The 60-min OT started immediately after the administration of low-frequency rTMS (interval time, 3.8 ± 1.0 min). The OT was administered by experienced occupational therapists on a one-to-one basis, and the program was tailored to fit each patient based on the motor function of the affected upper limb and their lifestyle (e.g., occupation, household work, interest). At the start of the intervention, patients were asked to identify daily activities that they wanted to improve, provided patients had sufficient movement in their affected upper limb to attempt the functional tasks, such as writing letters by pencils, using chopsticks to pick up small objects, pinching coins, and combing hair. For patients who did not have sufficient movement in their affected upper limb to practice the tasks, the therapists assisted the patients by guiding the limb through the tasks. All patients were given passive range of motion (P-ROM) exercises to facilitate mobility and decrease spasticity.

### 2.3 Outcome measures

All outcome measurements were performed at baseline (Baseline) and at the end of the intervention (Post). The primary outcome measure was the 14-item Fugl-Meyer assessment (FMA) motor scores for the distal upper limb (forearm supination/protonation with elbow at 0°; forearm supination/protonation with elbow at 90° and shoulder at 0°; and all items of the wrist and fingers; all items were measured with a score range of 0**–**28) [19,20]. Secondary outcome measures included quantitative evaluation of manual dexterity (modified 10-s tests) [21], the Motor Activity Log (MAL) Amount of Use (AOU) and Quality of Movement (QOM) scores [22], P-ROM of the finger joint and wrist joint extensions/flexions, and the modified Ashworth scale (MAS) of the finger and wrist extensors/flexors [23]. In the modified 10-s tests, patients performed four types of hand-finger movements (grip and release, individual finger movement, hand protonation and supination, and finger tapping) as many times as possible within 10 s. The score of the modified 10-s tests was defined as the sum of all repeated movements. The P-ROM score of the finger joint extensions or flexions was calculated as an average extension or flexion values of metacarpophalangeal joints II–V. Similarly, the MAS score of the finger extensors or flexors was calculated as the average extensor or flexor values of metacarpophalangeal joints II–V.

The MEP of the FDI on the affected side was also measured at Baseline and Post. MEP was elicited by TMS with 110% of the intensity of rMT. In each block, 15 stimuli were applied to each patient with an interval of 5–6 s between stimuli. The MEP was amplified, band-pass filtered (10–5000 Hz), and digitized at a rate of 10 kHz using a Neuropack MEB-9400 system (Nihon Kohden, Tokyo, Japan). Trials containing significant artifacts were visually inspected and removed, and the peak-to-peak MEP amplitude was measured and averaged in each block. At least 10 trials were included in the analysis for each block.

### 2.4 Statistical analysis

All analyses were performed using Microsoft Excel 2016 (Microsoft, Redmond, WA, USA) and SPSS version 23 (IBM Corp., Armonk, NY, USA). As multiple comparisons were made, Bonferroni corrections were applied to correct significance values. The results were considered significant at p < 0.05.

## 3 Results

The scheduled intervention protocol was completed by all 40 patients. None of the patients experienced any adverse events during the study period.

Baseline characteristics were compared using chi-square test between the mild and severe groups, and they were not significantly different, except for a higher rate of hemorrhage in the severe group (p = 0.010, Table 1). The median time since stroke was 1243 days.

Normal Gaussian distribution of the data was evaluated using Shapiro-Wilk test and observed only for the FMA scores (p = 0.15). To analyze the effects of low-frequency rTMS combined with OT on the FMA scores, a two-way repeated-measures analysis of variance was performed with time (Baseline and Post) and severity (mild and severe) as factors. In case of significant effects, post-hoc analyses were performed with Student's paired sample *t*-test. Other data were analyzed with Wilcoxon's signed-rank test. Data are presented as mean ± standard deviation or median [interquartile range].

### 3.1 FMA scores

**Fig. 2:**
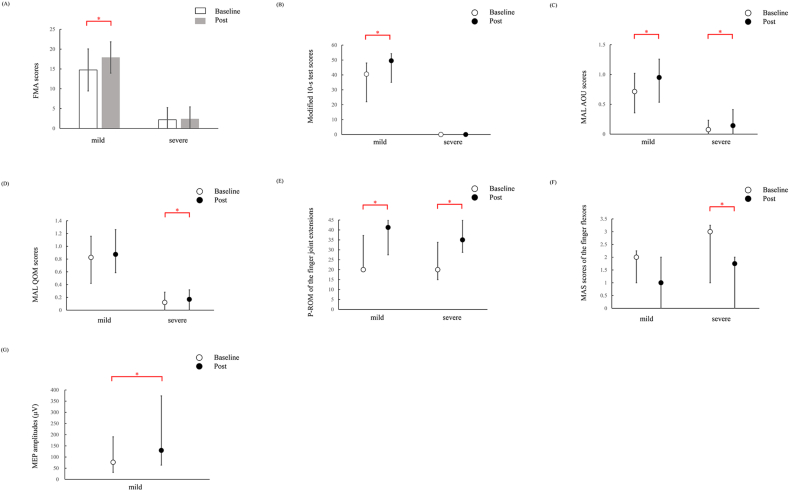
Patient outcome measures before (Baseline) and after intervention (Post). (A) Fugl-Meyer assessment (FMA) scores. (B) Modified 10-s test scores. (C) Motor Activity Log (MAL) Amount of Use (AOU) scores. (D) MAL Quality of Movement (QOM) scores. (E) Passive range of motion (P-ROM) of the finger joint extensions. (F) Modified Ashworth scale (MAS) scores of the finger flexors. (G) Motor evoked potential (MEP) amplitudes. Bars are mean values and error bars are standard deviations (A). Circles are median values and error bars are interquartile ranges (B–G). Asterisks indicate statistically significant differences.

The FMA scores of the mild group were 14.75 ± 5.29 at Baseline and 17.90 ± 3.95 at Post, whereas those of the severe group were 2.20 ± 3.05 at Baseline and 2.40 ± 3.03 at Post (Fig. 2A). The statistical analysis showed a significant main effect for both time (F_1,19_ = 51.66, p < 0.001, η_p_^2^ = 0.73) and severity (F_1,19_ = 172.33, p < 0.001, η_p_^2^ = 0.90), as well as a significant interaction between time and severity (F_1,19_ = 42.98, p < 0.001, η_p_^2^ = 0.69). Post-hoc analyses demonstrated that the FMA scores increased significantly from Baseline to Post in the mild group (p < 0.001), but not in the severe group (p = 0.33).

### 3.2 Modified 10-s tests

The mild group showed a significant increase in the modified 10-s tests scores from 40.5 [22–48] to 49.5 [35–54.25] (p < 0.001). The severe group showed no significant change after the intervention (Baseline, 0 [0–0]; Post, 0 [0–1]; p = 0.051) (Fig. 2B).

### 3.3 MAL scores

The mild group showed a significant increase in the AOU scores from 0.71 [0.36–1.02] to 0.95 [0.54–1.26] (p = 0.023). The severe group also showed a significant increase from 0.08 [0–0.23] to 0.14 [0–0.41] (p = 0.007) (Fig. 2C). The QOM scores did not change significantly after the intervention in the mild group (Baseline, 0.83 [0.42–1.16]; Post, 0.87 [0.59–1.26]; p = 0.056). However, the severe group showed a significant increase from 0.12 [0–0.28] to 0.17 [0–0.32] (p = 0.023) (Fig. 2D).

### 3.4 P-ROM scores

The P-ROM values for finger joint flexions did not change significantly after the intervention in either the mild group (Baseline, 90° ([90°–90°]; Post, 90° [90°–90°]; p > 1) or the severe group (Baseline, 90° [80°–90°]; Post, 90° [80°–90°]; p = 0.67). The P-ROM values for finger joint extensions increased significantly in the mild group from 20° [20°–37.19°] to 41.25° [27.5°–45°] (p = 0.017). The severe group also showed a significant increase from 20° [15°–33.75°] to 35° [28.75°–45°] (p = 0.005) (Fig. 2E). However, the P-ROM values for wrist joint flexion did not change significantly in either the mild group (Baseline, 85° [70°–90°]; Post, 90° [84.5°–90°]; p = 0.26) or the severe group (Baseline, 80° [70°–90°]; Post, 90° [77.5°–90°]; p = 0.16). The P-ROM values for wrist joint extension also did not change significantly in either the mild group (Baseline, 70° [50°–70°]; Post, 70° [63.75°–70°]; p = 0.41) or the severe group (Baseline, 50° [50°–62.50°]; Post, 60° [50°–70°]; p = 0.08).

### 3.5 MAS scores

The MAS values for finger flexors did not change significantly after the intervention in the mild group (Baseline, 2 [1–2.25]; Post, 1 [0–2]; p = 0.16). The severe group showed a significant decrease in the MAS values from 3 [1–3.25] to 1.75 [0–2] (p = 0.007) (Fig. 2F). The MAS values for finger extensors did not change significantly in either the mild group (Baseline, 0 [0–0]; 0 [0–0]; p > 1) or the severe group (Baseline, 0 [0–0]; Post, 0 [0–0]; p > 1). The MAS values for wrist flexors did not change significantly in either the mild group (Baseline, 1 [0–2]; Post, 0.5 [0–1]; p = 0.47) or the severe group (Baseline, 2 [0–3]; Post, 1 [0–3]; p = 0.27); neither did the MAS values for wrist extensors change significantly in either the mild group (Baseline, 0 [0–1]; Post, 0 [0–0.25]; p > 1) or the severe group (Baseline, 0 [0–1]; Post, 0 [0–1]; p > 1).

### 3.6 MEP values

The mild group showed a significant increase in MEP from 76.4 [31.6–190.5] to 129.7 [63.9–373.4] μV (p = 0.001) (Fig. 2G). In the severe group, MEP was not detected in any patient at either Baseline or Post.

## 4 Discussion

This study showed that low-frequency rTMS applied to the contralesional M1 in combination with OT improved upper limb use, quality of movement, the P-ROM of the finger joint extensions, and the MAS of the finger flexors even in chronic stroke patients with severe upper limb hemiparesis. In addition, we confirmed that this strategy could improve upper limb motor recovery as measured with the FMA scores, manual dexterity, upper limb use, the P-ROM of the finger joint extensions, and MEP in mild patients. The latter finding is consistent with previous studies [[9], [10], [11], [12], [13]]. The results of the NICHE trial, a randomized, blinded, sham-controlled clinical trial with a high number of participants, does not support the beneficial effects of low-frequency rTMS applied to the contralesional M1 compared with a control group [24]. This may be due to the difference of the protocols among studies. First, the NICHE trial included patients with <6 months since stroke onset. These patients may have had the spontaneous recovery; thus, the beneficial effects of the intervention are unclear. Second, in the NICHE trial, the treatment group received 900 TMS pulses at 110% rMT per session, whereas in our study, the treatment group received 1300 TMS pulses at 90% rMT per session. Furthermore, in the NICHE trial, the treatment group received 18 treatment sessions over 3 weeks, whereas in our study, the treatment group received 22 treatment sessions over 12 consecutive days. The differences in these stimulation parameters may have resulted in the differences in outcomes mentioned above.

Regarding the mechanism of functional recovery, one possibility is the improvement of the corticospinal tract (CST) from the ipsilesional M1 (the ipsilesional CST) in these patients. Low-frequency rTMS applied to the contralesional M1 reduces the abnormally enhanced IHI from the contralesional M1 to the ipsilesional M1 by inhibiting the excitability of the contralesional M1, which might enhance the excitability of the ipsilesional CST [10]. Although the biophysical mechanisms of low-frequency rTMS is still not completely understood, it is suggested that low-frequency rTMS can induce synaptic plasticity like long-term depression [25]. Connectivity analyses in a functional MRI study revealed that low-frequency rTMS applied to the contralesional M1 led to a decrease in the IHI from the contralesional M1 to the ipsilesional M1 [26]. Cathodal transcranial direct current stimulation, which suppresses cortical excitability, of the contralesional M1 also promotes motor function recovery in stroke patients [27]. Downregulating the excitability of the contralesional M1 based on the interhemispheric competition model [8] has been an effective strategy for motor function recovery in chronic stroke patients with mild hemiparesis.

However, none of the patients with severe hemiparesis had any MEP response to the TMS applied to the ipsilesional M1, which is consistent with a previous study showing that the degree of motor deficit in patients with chronic stroke was highly dependent on the proportion of the CST affected by the lesion [28]. As the recovery of upper limb impairment after stroke depends on the integrity of the ipsilesional CST [29,30], it is expected that motor function recovery might be limited in patients with severe damage to the ipsilesional CST, even after intensive rehabilitation such as low-frequency rTMS combined with OT.

Here, we discovered improvement in upper limb use and quality of movement for severe stroke patients, indicating functional recovery in everyday life. Our results suggest that this clinical improvement in the real world may not be directly related to the enhanced excitability of the ipsilesional CST but may be associated with the reduction of upper limb spasticity.

Improvement in spasticity may allow increased use of the upper limb among patients with severe stroke and improve their quality of movement. Spasticity is a motor disorder characterized by hyperexcitability of the stretch reflex [31,32], and can induce pain, ankylosis, or muscle weakness. Although the underlying mechanism of spasticity remains poorly understood, post-stroke spasticity results from an imbalance of the supraspinal inhibitory and excitatory tracts descending to the spinal cord [33]. Evidence from animal and human studies shows that descending tracts other than CST, such as the reticulospinal tract, are largely involved in the mechanism of post-stroke spasticity [34,35].

We found no improvement in MAS values in patients with mild stroke. Regarding the MAS values at Baseline in patients with mild stroke, the most and least affected muscles were finger flexors (2 [1–2.25]) and finger extensors (0 [0–0]), respectively. As the patients with mild stroke had slight or no spasticity at Baseline, spasticity may not improve any further after the intervention. In patients with mild stroke, the enhanced excitability of the ipsilesional CST rather than the improvement in spasticity may have a significant contribution to upper limb motor recovery.

In this prospective, non-controlled, pilot study, low-frequency rTMS combined with OT improved spasticity in patients with severe upper limb hemiparesis. This may be due to the functional recovery of descending tracts other than CST via therapeutic intervention. Improvements in spasticity were observed only in finger movements (finger joint flexion or extensions), but not in wrist movements, suggesting a somatotopic effect of rTMS that targeted the M1 of the finger muscle. For this reason, the low-frequency rTMS with OT in our protocol might be particularly suitable for ameliorating spasticity of the metacarpophalangeal joints [36].

### 4.1 Study limitations

First, because this is a pilot study, it lacked a control group. The observed effects of our intervention may have resulted from non-specific aspects of the treatment. However, the possibility is unlikely because we included patients with chronic stroke whose symptoms had not improved with conventional rehabilitation. This speculation is supported by the fact that the observed benefits in mild patients were consistent with those in previous studies [[9], [10], [11], [12], [13]]. Second, the effect of low-frequency rTMS without OT was not investigated. The combination of non-invasive brain stimulations and conventional rehabilitative therapies can have a synergistic effect on the functional recovery compared with either therapy alone. The synergistic effect may result from the use-dependent plasticity induced by repetitive training [37]. To address both of these limitations, a future study should investigate the synergistic effect of low-frequency rTMS and OT by comparing the therapeutic effects of OT, those of low-frequency rTMS, and those of low-frequency rTMS combined with OT. Third, as we could not stop or adjust the patients’ medication prior to the study for ethical reasons, there was heterogeneity among in medication use among our patients, which might have influenced the results.

## 5 Conclusions

We found that low-frequency rTMS applied to the contralesional M1 in combination with OT was effective for the rehabilitation of chronic stroke patients with severe upper limb hemiparesis. As the ipsilesional CST was severely damaged in patients with severe hemiparesis, the anti-spastic effect of this intervention strategy might lead to an increase in upper limb use and improve quality of movement.

## Author contribution statement

Yoshihiro Yukawa: Conceived and designed the experiments; Performed the experiments; Analyzed and interpreted the data; Wrote the paper.

Sumiya Shibata, Tatsuya Mima: Conceived and designed the experiments; Analyzed and interpreted the data; Contributed reagents, materials, analysis tools or data; Wrote the paper.

Satoko Koganemaru: Conceived and designed the experiments; Analyzed and interpreted the data.

Masatoshi Minakuchi, Ryota Shimomura: Performed the experiments, Analyzed and interpreted the data.

Kazuhito Nakamura: Conceived and designed the experiments; Contributed reagents, materials, analysis tools or data.

## Source of funding

This study was supported by Grants-in-Aid for Scientific Research (KAKENHI) [grant number 19H01091 (T.M.), 22H04788 (T.M.), and 23H00459 (T.M.)] from the 10.13039/501100001691Japan Society for the Promotion of Science.

## Data availability statement

Data will be made available on request.

## Declaration of competing interest

The authors declare the following financial interests/personal relationships which may be considered as potential competing interests:

Department of Regenerative Systems Neuroscience, Graduate school of Medicine 10.13039/501100005683Kyoto University is an endowed department funded by Kodama Foundation. - S.K.
